# *Mycobacterium tuberculosis*: escape room world champion

**DOI:** 10.1186/s41479-019-0060-2

**Published:** 2019-03-24

**Authors:** Thuc Anh Nguyen, Sophie Croon, Ger Rijkers

**Affiliations:** 1 Department of Sciences, University College Roosevelt, P.O. Box 94, 4330 Middelburg, AB Netherlands; 2 Laboratory for Medical Microbiology and Immunology, St Elisabeth Hospital, Tilburg, Netherlands

An escape room is a physical adventure game in which the players have to solve problems and develop a strategy to leave their confined physical space. In March 2017, Budapest hosted the first escape room championships, in which 22 countries participated; Slovenia won. If this championship would have taken place in the microbiological world, the winner probably would have been *Mycobacterium tuberculosis* (*M. tuberculosis*).

*M. tuberculosis* is the causative micro-organism of the most widespread infectious disease tuberculosis (TB). *M. tuberculosis* can be transmitted via air droplets into the lungs, where it is phagocytosed by alveolar macrophages. Normally, a macrophage would be able kill and digest the pathogen by fusing the phagocysome with a lysosome. However, in the case of *M. tuberculosis*, the bacterium can escape this lysosomal fusion which allows the bacterium to replicate and grow intracellularly in the phagocyte [1]. Because lysosomes contain a large arsenal of bactericidal enzymes, the prevention or postponement of fusion with the phagosome thus increases the chance of survival of the bacterium. Ironically, this type of host defense mechanism, phagocytosis, is beneficial to *M. tuberculosis* and other intracellularly growing bacteria such as Mycobacteria, Legionella and Salmonella species and *Listeria monocytogenes*; because by staying within the phagocytic cell, the microorganism is shielded from important components of the immune system including complement and antibodies. They extract the essential nutrients and minerals (including iron) directly from the host cell for their own growth, but in order to survive the intracellular microorganisms has to use ‘stealth’ techniques to escape bactericidal mechanisms of phagocytic cells.

**Fig. 1:**
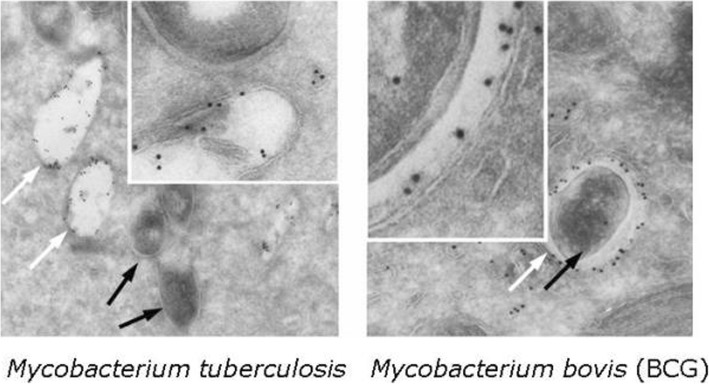
*Mycobacterium tuberculosis* breaks out of lysosomes. The left electron microscopic image shows *M. tuberculosis* bacteria (black arrows) in the cytoplasm of an infected dendritic cell. The remaining empty phagolysosomes (white arrows) have been stained with gold labeled antibodies against LAMP1 (lysosome associated membrane protein), a protein specifically for lysosomes Colloidal gold particles (visible as black dots) show the location of antibody binding (see also the inset on the photo). In the right figure a dendritic cell is infected with another mycobacterium strain, *M. bovis*. This is the mycobacterium from the BCG vaccine (Bacille Calmette Guérin) against tuberculosis. This mycobacterium remains within the lysosome. (Further details in reference [2])

As indicated above, *M. tuberculosis* can be taken up in lysosomes but after two to four days it can break out of the phagolysosome escaping its digestion and evades into the cytoplasm (Fig. 1) [2, 3]. Moreover, the characteristic of *M. tuberculosis* as an escape artist can be further shown by its ability to disseminate from the primary site of infection to peripheral lymph nodes and blood stream [4]. It thus now becomes apparent that the multiple levels of escape mechanisms from the host immune system makes *M. tuberculosis* an extremely pathogenic bacteria.

The current treatment of TB consist of a series of antibiotics which have to be taken for 6 months [5]. A full course of therapy (completion of treatment) is however determined more accurately by the total number of doses taken, not solely by the duration of therapy. For example, the “6-month” daily regimen (given 7 days/week) should consist of at least 182 doses of Isoniazid and Rifampin, and 56 doses of Pyrazinamide [6]. Antibiotic therapy hasn’t changed much because only one TB drug has entered the clinical practice in the past 40 years. Also in terms of prevention of TB progress is slow. The BCG vaccine has been in use for almost a hundred years, but unfortunately only has a limited effect in preventing tuberculosis [7]. Thus TB remains one of the deadliest infections known to man, causing 10.0 million infections and 1.6 million deaths in 2017 [8]. Combining these figures with the increased rate of infections by multidrug resistant strains, which already account for 50% of infections in some countries [9], means that a more effective vaccine is urgently needed. BCG has shown to protect vaccinees against deadly disseminated forms of TB including life threatening TB meningitis. However, at a later stage of infection when *M. tuberculosis*, BCG becomes ineffective [10]. While BCG mainly induces a cellular immune response, recent research now also focuses on development of vaccines inducing a humoral response, with antibodies against α-glucan, arabinomannan and lapidated arabinomannan [11]. There is a clear need for standardization of experimental designs with aligning of end-points which should facilitate the development of new vaccines [12]. Vaccine candidates, such as the ones mentioned above, could be combined in recombinant viral vectors [13]. In the development of better Mycobacterial vaccines it should also be kept in mind that a supraoptimal immune response may lead to considerable immunopathology [14, 15].

Although *M. tuberculosis* may be successful at escaping the phagolysosome, it is important that it does not escape our attention. Millions of patients are affected each year, and fully effective vaccines have not yet been developed. In order for this disease to stay on the radar, there is an annual World Tuberculosis Day on March 24. During this day, special attention is given to the victims of this infection, and to promote research leading to a better future for those that are currently infected, and those that will be infected in the future. Together, these efforts hopefully succeed in eradication of this disease, so that in the future it will fail to escape our attention once and for all.
